# High levels of incidental physical activity are positively associated with cognition and EEG activity in aging

**DOI:** 10.1371/journal.pone.0191561

**Published:** 2018-01-25

**Authors:** Javier Sanchez-Lopez, Juan Silva-Pereyra, Thalía Fernández, Graciela C. Alatorre-Cruz, Susana A. Castro-Chavira, Mauricio González-López, Sergio M. Sánchez-Moguel

**Affiliations:** 1 Departamento de Neurobiología Conductual y Cognitiva, Instituto de Neurobiología, Universidad Nacional Autónoma de México, Querétaro, México; 2 Department of Neurosciences, Biomedicine and Movement Sciences, University of Verona, Verona, Italy; 3 Facultad de Estudios Superiores Iztacala, Universidad Nacional Autónoma de México, Estado de México, México; 4 Institutt for psykologi, Det helsevitenskapelige fakultet, Universitetet i Tromsø –Norges arktiske universitet, Tromsø, Norway; 5 Escuela Superior de Atotonilco de Tula, Universidad Autónoma del Estado deHidalgo, Hidalgo, México; University of Florida, UNITED STATES

## Abstract

High levels of physical activity seem to positively influence health and cognition across the lifespan. Several studies have found that aerobic exercise enhances cognition and likely prevents cognitive decline in the elderly. Nevertheless, the association of incidental physical activity (IPA) with health and cognition during aging has not been studied. Thus, the aim of this study was to evaluate the association of IPA level with cognitive functions and resting electroencephalogram (EEG) in healthy old participants. Participants (n = 97) with normal scores on psychometric and neuropsychological tests and normal values in blood analyses were included. A cluster analysis based on the scores of the Yale Physical Activity Scale (YPAS) allowed the formation of two groups: active, with high levels of IPA, and passive, with low levels of IPA. Eyes-closed resting EEG was recorded from the participants; the fast Fourier transform was used offline to calculate absolute power (AP), relative power (RP), and mean frequency (MF) measures. There were no differences in socioeconomic status, cognitive reserve, general cognitive status, or lipid and TSH profiles between the groups. The results of cognitive tests revealed significant differences in the performance variables of the WAIS scores (p = .015), with advantages for the active group. The resting EEG exhibited significantly slower activity involving the frontal, central, and temporal regions in the passive group (p < .05). Specifically, higher delta RP (F7, T3), lower delta MF (F4, C4, T4, T6, Fz, Cz), higher theta AP (C4), higher theta RP (F4, C4, T3, Fz), lower alpha AP (F3, F7, T3), lower alpha RP (F7), and lower total MF (F3, F7, T3, T5, Fz) were found. Altogether, these results suggest that IPA induces a neuroprotective effect, which is reflected both in behavioral and electrophysiological variables during aging.

## Introduction

Aging is accompanied by a normal decline in important cognitive functions, such as processing speed, executive control, inhibition, working memory, and episodic memory [1]. These declines in the cognitive status of the elderly are associated with structural and functional modifications in the brain [2]. Different studies have found age-related changes in the resting electroencephalogram (EEG) and EEG during the performance of cognitive tasks [3–10]. According to Giaquinto & Nolfe [9], a slower resting EEG is characteristic of old age. This EEG slowing is evidenced by a decrease in frequency [3] and amplitude [4] of the occipital alpha rhythm, associated with a decrease in posterior alpha power; the appearance of dispersed theta waves, related to a diffuse increase in theta power [10]; and the occasional presence of delta waves, mainly in the temporal sites [10]. In contrast with the idea that these changes are the result of normal aging, some authors [3] have proposed that these changes may be a result of an ongoing subclinical pathological process. In fact, a large increase in the theta activity may predict cognitive impairment in the elderly [6]. A recent multimodal study in the elderly using both structural magnetic resonance imaging (MRI) and EEG found associations among the activity in the theta frequency band, cortical thickness, and performance in a visuospatial task [8]. Furthermore, these aforementioned EEG changes are exacerbated in patients with mild cognitive impairment and dementia [11]. Besides these results, some previous studies have also reported increased alpha activity in frontal areas [5], which might be related to compensatory mechanisms against cognitive decline [7].

Several studies have suggested that lifestyle significantly influences overall health, particularly cognition, during aging [12] in association with modifications in the recruitment of brain networks [13]. As a component of the lifestyle, physical activity seems to positively influence several body systems, such as the respiratory, circulatory and muscular systems [14], producing variations in hematological [15] and hormonal parameters [16] and improving cognitive function [1,17]. Furthermore, the increase in the level of physical activity, acting as a neuroprotective mechanism, may reduce declines in cognitive functions and offer protection against dementia during normal aging [18]. A study by Barnes and colleagues in old adults [19] reported that the level of aerobic fitness (i.e., the capacity of the cardiorespiratory system to use oxygen), which is positively related to the practice of aerobic physical activity, predicts performance on a cognitive task involving working memory, processing speed, attention, and general mental functioning up to six years later. In a meta-analysis, Colcombe and Kramer [20] reported similar results in seniors, where aerobic fitness was strongly associated with executive processes and performance in controlled, spatial, and speed tasks. Therefore, the processes that undergo age-related decline seem to be also enhanced by aerobic physical activity; for this reason, several interventions in the elderly are based on programs of physical activity aiming to provide physical and cognitive wellness among this population.

There are two main approaches to the study of the associations of physical activity with health and cognition: a) the effects of structured physical activity either acute or long-term [20–22], which is the most common approach, and b) the association of incidental physical activity (IPA) with fitness quality [e.g., 18,22]. IPA, as opposed to structured physical activity, is the result of unstructured daily activities, such as working, housekeeping, transportation, leisure, etc. [1,12,23].

Our study, which pertains to the second approach, is focused on the association of IPA with cognition and brain electrical activity. Since IPA comprises daily activities, it involves an ecological environment that is easily accessed by everyone, including seniors who less frequently practice structured physical activity [24]. To our knowledge, there are no previous studies that relate IPA to EEG and cognition. Nevertheless, few electrophysiological studies have investigated the relationship between structured physical activity, cognition, and aging, and even fewer have examined the changes in resting EEG associated with physical activity in the elderly [25–31]. Most of these studies included populations of old adults, although not all participants coursed with normal aging [e.g., 25,28]. In general, these studies considered structured physical activity, and the time of training ranged from 15 min to eight weeks. A common feature of most of the studies is an increase in alpha absolute power (AP) and/or alpha relative power (RP) of the resting EEG as a consequence of the training [25,27,28,31]. A delta decrease [27] and a theta [27,31] and beta [25,27] increase were also observed. The training effects were more evident in younger than in old adults [28] and occurred mainly in frontal channels.

Therefore, considering that a) age-related cognitive decline is identified by a decay in several cognitive processes, which are related to a lower-frequency EEG [e.g., 31]; b) that structured physical activity promotes positive effects on cognition, EEG [e.g., 26] and physiological measurements [14]; and c) that IPA and structured physical activity have similar physiological effects [32], the main purpose of this study was to evaluate the relationship of the IPA level with cognitive function and resting EEG; as a secondary objective, we proposed to investigate the association of IPA with anthropometrics and blood variables. We expected that if IPA and structured physical activity have similar neuroprotective mechanisms, IPA will also be positively associated with cognition and quantitative EEG (QEEG) (i.e., seniors with higher levels of IPA will exhibit lower delta and theta and higher alpha and beta activities).

## Materials and methods

### Participants

Through a nonprobability judgment sampling, 114 elderly individuals were invited to participate in the study. After an initial screening based on the inclusion/exclusion criteria (described below), this study involved 97 participants aged 60 years and older (mean age = 66.80 years old, SD = 4.30); there were 64 female and 33 male participants. The inclusion criteria considered participants, who at least completed middle school (mean of years of schooling = 15.65, SD = 4.09) and lacked cognitive impairments or clinical symptoms of cognitive deterioration. None of them had major socioeconomic disadvantages (The Mexican Association of Marketing Research and Public Opinion Agencies; AMAI 8 x 7 questionnaire) or evidence of depression as indirectly measured by the Quality of Life Enjoyment and Satisfaction Questionnaire (Q-LES-Q), which is a self-reported measure of satisfaction in seven daily-functioning domains: bodily health, subjective feelings, job, housekeeping, leisure activities, social relationships, and general satisfaction. Responses were scored on a 5-point scale, from “Never” to “All the time”, with higher scores implying more enjoyment and satisfaction [33]. The scores of participants in the Global Deterioration Scale (GDS) [34] did not show signs of cognitive decline. All participants had normal scores on the brief neuropsychological test battery in Spanish (Neuropsi) [35], a standardized test for the Mexican population with norms by age and educational level. This neuropsychological battery briefly assesses a wide spectrum of cognitive functions, including orientation, attention, memory, language, visuoperceptual abilities, and executive functions.

Participants with neurological or psychiatric disorders were excluded from this study. In addition, individuals with untreated anemia or metabolic, cardiac or thyroid diseases were not included.

The Ethical Committee of the Institute of Neurobiology at the National Autonomous University of Mexico approved this study. The main incentive for volunteers was that free access to their results of the clinical screening was provided. All volunteers signed informed consent forms. The entire study was conducted in the Psychophysiology Laboratory of the Institute of Neurobiology at the National Autonomous University of Mexico, in Juriquilla, Queretaro, Mexico.

### Phase one: Participant classification according to physical activity level

#### Physical activity instrument

To know the level of physical activity, all participants completed the Yale Physical Activity Survey (YPAS) in its Spanish version [36]. The YPAS assesses physical activity in adults aged 60 and older. This questionnaire has shown acceptable reliability (test-retest R from .19 to .66, p < .05) and validity (Spearman correlations with anthropometric and physiological measures from .20 to .29, p < .05) in old adults [36] and diverse patient populations across different cultures [e.g., 36,37,38]. The YPAS is divided into two parts. The first part asks the participants about the time spent on specific activities across different domains (i.e., housework, working, yard work, care-taking and leisure activities). Three values are obtained for each specific activity: time (minutes/week), energy expenditure in MET (Metabolic Equivalent of Task-MET-*time) and energy expenditure (MET*time*weight), measured in kilocalories; finally, a sum of the values for each domain and a total sum are calculated. The second part of the questionnaire asks about the frequency and time spent in vigorous activities, leisurely walking, standing, moving and sitting. The partial indices are calculated by multiplying the frequency score by the duration score for each specific activity and multiplying again by a weighting factor. The total index is obtained when partial indices are added.

#### Data analysis for group formation

**Fig 1:**
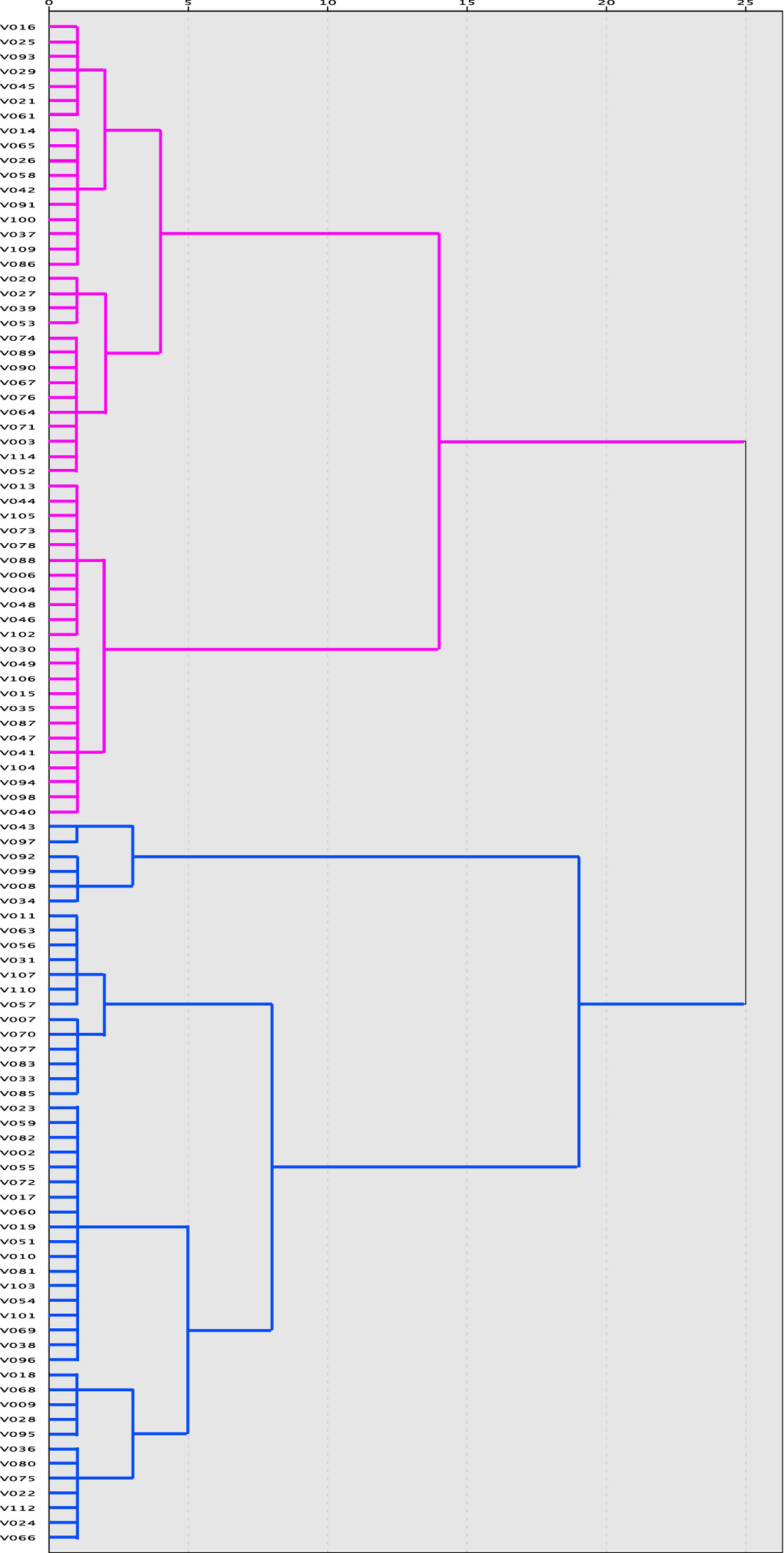
Dendrogram resulting from a hierarchical cluster analysis. Two clusters were observed: one with high levels of IPA (cyan color) and other with low levels of IPA (magenta color). After to remove participants with extreme values in age into each cluster, two homogeneous groups were obtained (Active Elderly: n = 48 and Passive Elderly: n = 49) for the further analyses.

To perform further analysis on cognitive, physiological, and electroencephalographic variables, a hierarchical cluster analysis was applied to identify possible homogeneous subgroups of old adults according to the variables from the YPAS. The total kcal/week, vigorous activity index, and moving index were used for a cluster analysis, which was performed using the Ward method with a measure of squared Euclidean distance [39], since this distance is sensitive to the variable metrics, standardized Z scores were used. A visual inspection of the dendrogram revealed two independent clusters that were equal in size and with different characteristics (see Fig 1). Finally, the following two groups were obtained: Active Elderly: n = 48 (31 females) and Passive Elderly: n = 49 (33 females).

#### Sociodemographic description and group comparisons for possible confounding variables

To rule out the possible differences between groups not related to physical activity, the groups were compared considering the following variables: cognitive reserve, age, years of schooling, and gender.

Cognitive Reserve is an important factor to consider in studies where cognition and brain activity are implicated because better cognitive performances and changes in brain activity pattern when have been observed cognitive reserve proxy measures are high. In a similar approximation to assess cognitive reserve as reported previously [40], a questionnaire was applied to obtain information about the activities of daily living. The Cognitive Reserve questionnaire (CRq) includes items that assess a diverse variety of activities, such as reading, playing a musical instrument, collecting things, practicing other language or dialects, traveling, or taking part in sports. The CRq is organized into four categories: everyday activities (EA), leisure activities (LA), social environment (SE), and training information (TI). A two-way ANOVA was performed to analyze differences between the groups regarding the different CRq categories. Group (active versus passive) was included as a between-subjects factor, and CRq (EA, TI, LA, and SE) was included as a within-subjects factor. The results of the ANOVA did not showed significant main effect of group (F < 1), or a significant group by CRq interaction (F < 1). As shown in Table 1, no significant differences between the groups were observed in years of schooling, gender, and socioeconomic level; only differences in age were found. Posterior statistical analyses were conducted on variables standardized by age or by using ANCOVA including age as a covariate.

**Table 1:** Socio-demographic information of the sample.

			t-student (95)			
	Mean (SD)				Confidence Interval 95%	
Variable	n = 48 Actives	n = 49 Passives	t	p-level	Inferior	Superior
Age	65.85 (3.80)	67.65 (4.51)	-2.12	.04	-3.48	-.12
Years of schooling	15.88 (4.17)	15.43 (3.97)	.54	.59	-1.19	2.09
F(n): M(n)	31:17	33:16	.08^a^	.77		
Socioeconomic level	51.11^b^	46.93^b^	1074.50^c^ (-.97)	.33		

Note

^a^ Chi^2^ value with 1 degree of freedom

^b^ Mean Rank

^c^ Mann-Whitney U (Z)

F: females; M: males

### Phase two: Comparisons between active and passive groups with respect to physical activity, cognition, EEG, and hematological variables

#### Physical activity analyses

Cluster analysis is an exploratory and descriptive method and not an inferential statistical tool. We forced the analysis to obtain two groups, but this does not mean that there was any significant difference between the groups across physical activity variables. So, once the two clusters were obtained, a mixed two-way ANCOVA was performed to assess differences between the groups in daily physical activity measured through energy expenditure in kilocalories and using age as a covariate. Group (actives and passives) was included as a between-subjects factor, and the YPAS domains (house work, work, yard work, care taking and leisure), as a within-subject factor. A mixed two-way ANCOVA was also performed to assess differences between the groups in frequency and time spent for every index of physical activity. Group was included as a between-subjects factor and physical activity indices (vigorous activity index, leisurely walking activity index, standing index, moving index and sitting index) as a within-subject factor. The Greenhouse- Geisser correction was applied to correct for violations of sphericity when there was more than one degree of freedom in the numerator. Tukey’s honest significant difference (HSD) post-hoc tests were completed after the ANCOVA.

#### Instruments for cognitive assessment

The Wechsler Adult Intelligence Scale in Spanish (WAIS-III-R) [41] was administered to the participants. WAIS-III-R is designed to measure intelligence, understood as IQ (normal or superior scores above 90), in participants between 16 and 89 years of age of any race, intellectual, educational and reading level, and any socioeconomic and cultural background. The scale is standardized, and measures of reliability (R from .88 to .97) and criterion validity with respect to the WAIS-III-R (R from .86 to .94) were obtained in the Mexican population [41]. It is administered individually and consists of 14 subtests: vocabulary, similarities, information, digit span, arithmetic, letter-number sequencing, comprehension, symbol search, coding, picture completion, block design, matrix reasoning and picture arrangement. By grouping the different subtests into a full-scale IQ, 2 sub-indices, verbal and performance IQs, and 4 secondary index scores, verbal comprehension index (VCI), working memory index (WMI), perceptual organization index (POI) and processing speed index (PSI), are obtained.

A series of mixed two-way ANOVAs were performed to analyze the differences between groups regarding different cognitive measurements from the WAIS-III-R. Group (Active and Passive) was included in the ANOVA model as a between-subjects factor, and domains were assessed using the WAIS-III-R full-scale and verbal and performance IQs or WAIS secondary indices (VCI, WMI, POI, and PSI) as a within-subjects factor. Two more mixed, two-way ANOVAs were also performed considering group as a between-subjects factor and 1) verbal IQ subscales (vocabulary, similarities, information, comprehension, arithmetic, digit span, and letter-number sequencing) as a within-subjects factor and 2) performance IQ subscales (picture completion, block design, matrix reasoning, digit symbol-coding, symbol search, and picture arrangement) as a within-subjects factor. The effect sizes (η^2^_p_) were reported. The Greenhouse-Geisser correction was applied to correct for violations of sphericity when there was more than one degree of freedom in the numerator (ε is reported). Tukey’s Honestly Significant Difference (HSD) method was used for post-hoc pairwise comparisons in the repeated-measure analyses.

#### EEG

Participants were seated in a comfortable chair in a dimly lit room. Digital resting EEG was recorded with eyes closed using a Medicid™ IV system (*Neuronic Mexicana*, *S*.*A*.; Mexico) and Track Walker TM v5.0 data system, for 30 min from the 19 channels of the 10–20 system (ElectroCap™, International Inc.; Eaton, Ohio) referenced to the linked earlobes (A1A2). The amplifier bandwidth was set between .50 and 50 Hz. All electrode impedances were at or below 10 kΩ, the sampling rate was 200 Hz, and the signal was amplified with a gain of 20,000. An expert electroencephalographer selected twenty-four artifact-free segments of 2.56 s by visual editing for quantitative analysis.

The QEEG analyses were performed off-line. The fast Fourier transform and cross-spectral matrices were calculated every .39 Hz; the following QEEG measures were calculated for each subject: 1) AP, 2) RP and 3) MF, within the delta (1.56–3.89 Hz), theta (3.90–7.50 Hz), alpha (7.51–12.50 Hz) and beta (12.51–19.15 Hz) frequency bands for each referential channel [6,11,42–44]; in addition, total MF was computed. The geometric power [45] of each individual was subtracted from his/her cross-spectral matrix to obtain the AP value in each band for each electrode; this correction reduces 42% of the variance that is not associated with physiological factors.

This software has a normative database [46,47] by age, and EEGs in the database were recorded in the 19 channels of the 10–20 system using A1A2 as a reference. When norms by age are used, the effect of age is removed. To obtain normative measures, 24 segments of 2.56 s were considered for analysis and bandwidths equal to those mentioned above. Using this normative database [46], Z values were obtained with the formula: Z = [X_i_ - μ] / σ, where X is the raw value of the *i*^*th*^ subject, and μ and σ are the mean value and the standard deviation, respectively, of the normal subjects of the same age as the *i*^*th*^ subject. In all, a total of 247 QEEG Z values were calculated for each subject.

Because of the multivariate nature of the EEG data, a non-parametric, multivariate permutation test [48] was used to explore whether there were differences between active and passive groups. The global test was performed using the permutation distribution of the maximum of the t-Student statistics. The distribution estimated by permutation techniques for *max t* was used to set significance levels that controlled the experiment-wise error for the simultaneous univariate comparisons. Thus, one can simultaneously test EEG differences for all the electrodes and avoid the inflation of type I errors [48]. AP, RP and MF for each frequency band were independently compared between the groups; MF was also calculated for the total frequency range between 1.56 and 19.14 Hz. In each case, a global probability (that simultaneously includes all electrodes) and 19 marginal probabilities (one per electrode) were reported in the results.

### Anthropometrics and blood analyses

Additionally, anthropometrics and blood analysis variables were compared between the groups by means of t-tests. Body mass index (BMI) was determined in each participant using BMI = Weight (kg) / Squared Height (m^2^). Blood samples were collected in the morning after an overnight fast from each subject. Blood biochemical analyses, including a lipid profile comprising total cholesterol, HDL, LDL, VLDL, and triglyceride and basal glucose levels, were determined for each patient. Hematic biometry and the level of thyroid-stimulating hormone (TSH) were also determined.

## Results

### Physical activity

**Fig 2:**
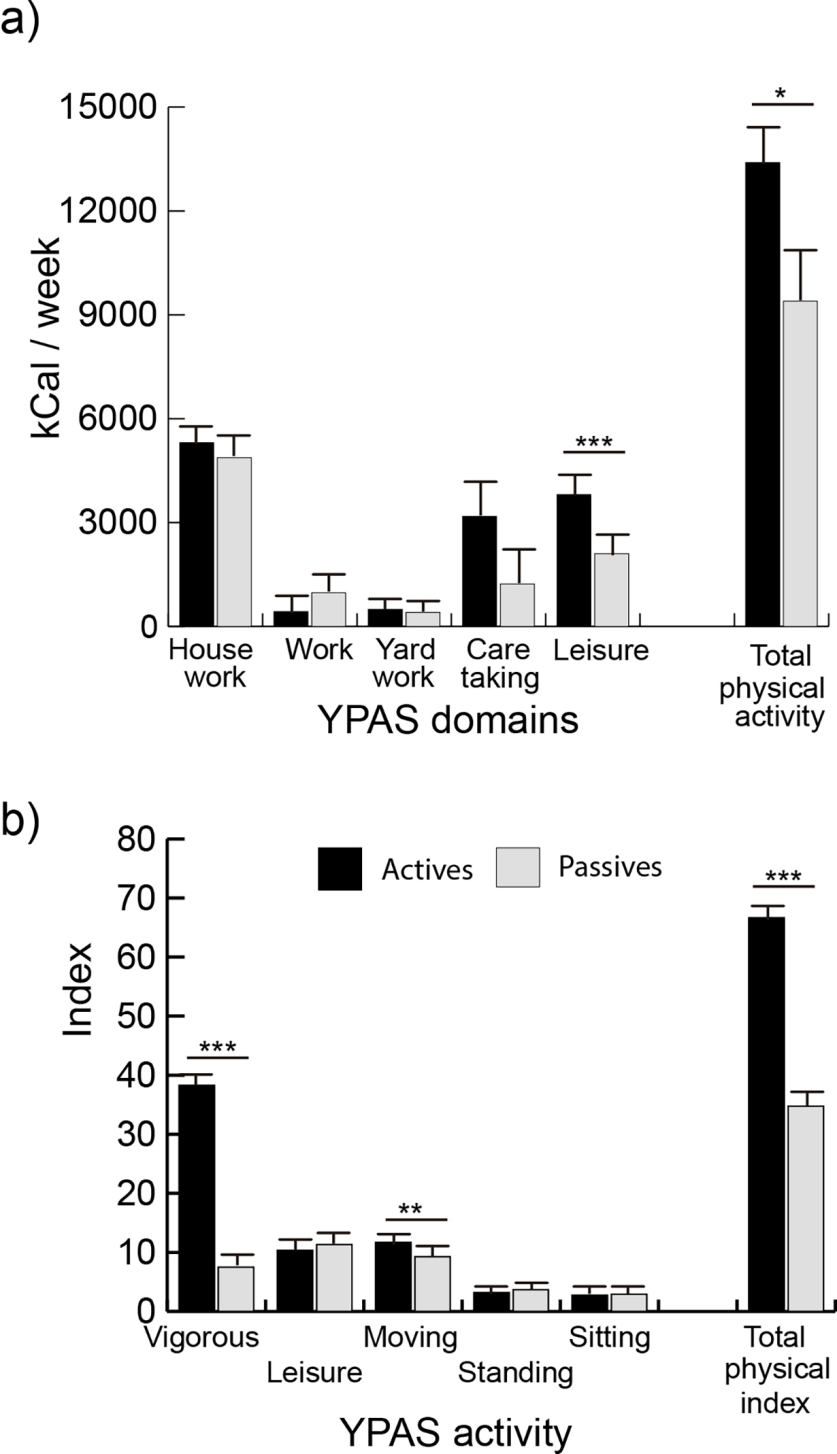
Post-hoc comparisons between the groups in the YPAS variables. * p < .05, ** p < .01, *** p < .001.

We compared actives *versus* passives in terms of kcal/week of the YPAS variables using age as a covariate. The ANCOVA showed a significant main effect of the group (F(1, 94) = 4.90, p = .03, η2p = .05), which indicates a greater kcal/week expenditure in actives than in passives. Significant differences were observed in the interaction group by physical activity (kcal/week) (F(4, 376) = 3.15, p = .04, ε = .57, η2p = .03). The multiple-comparison, post-hoc analyses are displayed in Fig 2A. ANCOVA regarding physical activity indices showed a significant main effect of group (F(1, 94) = 109.40, p < .0001, η2p = .54), indicating greater scores for active people than passives. There was also a significant group by physical activity indices effect (F(4, 376) = 83.50, p < .0001, ε = .45, η2p = .47). Fig 2B shows the post-hoc analyses results, where actives had greater scores than passives in vigorous and moving activity.

### WAIS

**Fig 3:**
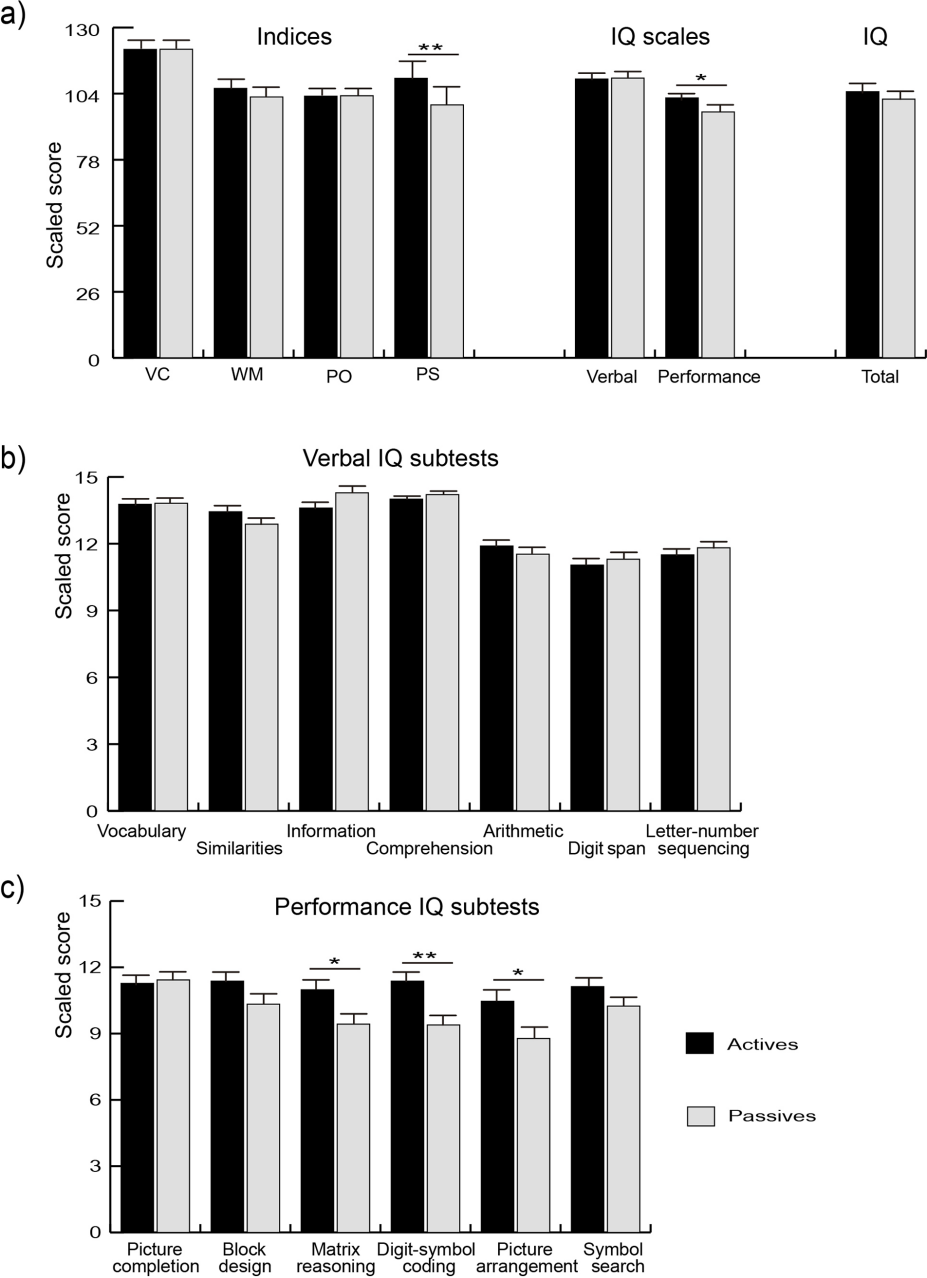
**Comparisons between active (n = 48) and passive (n = 49) groups for the WAIS-III-R variables:** a) Indices, IQ scales and total IQ; b) verbal WAIS subtests; and c) performance WAIS subtests; * p < .05; ** p < .01. IQ: Intelligence quotient; VCI: verbal comprehension index; WMI: working memory index; POI: perceptual organization index; PSI: processing speed index.

The ANOVA results showed no significant main effect of group (active versus passive; F(1, 95) = 2.23, p = .13, η^2^_p_ = .02), but an important significant group by IQ-subscale (verbal versus performance IQs) interaction (F(1, 95) = 8.98, p < .01, η^2^_p_ = .09) was found. These results mean that actives had higher performance IQ scores than passives, which is shown in Fig 3A. In terms of the secondary indices (VCI, WMI, POI and PSI), the ANOVA results showed no significant main effect of group (F(1, 95) = 3.1, p = .08, η^2^_p_ = .03), but a significant group by secondary-index interaction (F(3, 285) = 5.70, p < .01, η^2^_p_ = .05 ε G-G = .66). Fig 3A also displays greater scores of actives than those of passives in PSI.

Subtests belonging to the verbal IQ category were included as within-subject factors in one separate ANOVA (vocabulary, similarities, information, comprehension, arithmetic, digit span, and letter-number sequencing). The results showed no significant main effect of group (F < 1) nor group X verbal IQ subtests (F(6, 570) = 1.40, p = .24, η^2^_p_ = .02, ε = .77), as is shown in Fig 3B. The ANOVA with the performance IQ subtests (picture completion, block design, matrix reasoning, digit symbol-coding, symbol search and picture arrangement) included as within-subject factors revealed a significant main effect of group (F(1, 95) = 6.20, p = .02, η^2^_p_ = .06). Actives displayed larger scores than passives. There was a significant group by performance IQ subtests interaction (F(5, 475) = 3.53, p < .01, η^2^_p_ = .04, ε = .88), where actives displayed larger scores on matrix reasoning (MD = 1.6, p = .02), digit symbol-coding (MD = 1.99, p < .01), and picture arrangement (MD = 1.7, p = .01), but not in picture completion (MD = -.16, p = .77), block design (MD = 1.05, p = .09) or symbol search (MD = .88, p = .07), as is shown in Fig 3C.

### EEG

**Fig 4:**
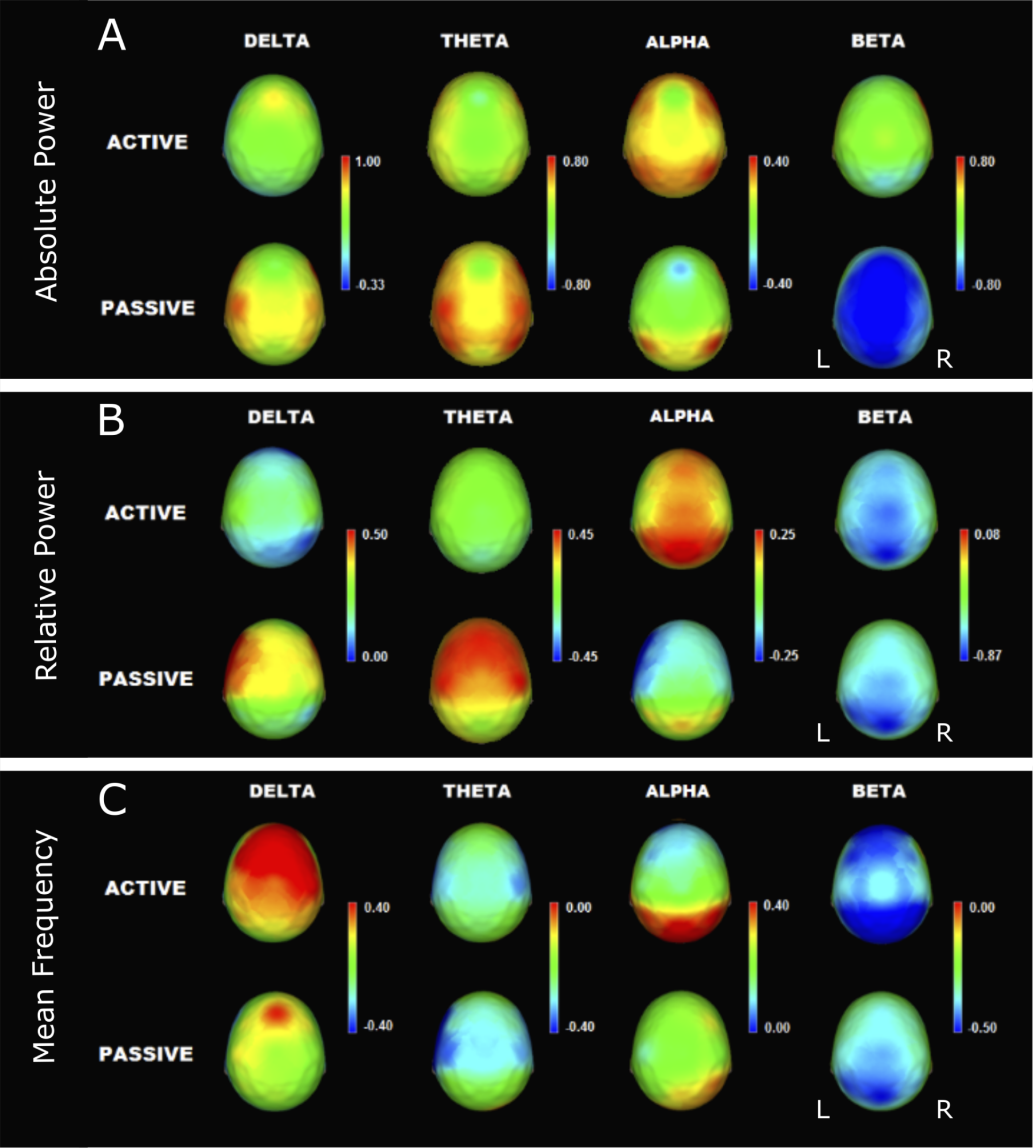
**Mean Z values of absolute power (A), relative power (B) and mean frequency (C) in the active (upper, n = 48) and passive (bottom, n = 49) groups.** Note that all values are within normal limits; however, the passive group has a slower EEG activity, which corresponds to higher power values in the delta and theta bands and lower power values in the alpha band. Mean frequency of the delta band indicates faster delta activity in the active group.

When the spectral EEG variables of the active group were compared with variables of the passive group, some differences were observed: a) less theta AP at C4 (p < .05) and more alpha AP at F3, F7 and T3 (p < .05); b) less delta RP (Global p = .04) at F7 and T3 (p < .05), less theta RP at F4, C4, T3 and Fz (p < .05), and more alpha RP at F7 (p < .05); and c) higher total and delta MF at F3, F7, T3, T5, Fz (p < .05) and at F4, C4, T4 T6 (p < .05), respectively. The results are shown in Table 2 and Fig 4.

**Table 2:** EEG spectral differences between the active and passive groups.

Measure	Frequency band	Active^a^ > Passives^b^		Passive^b^ > Actives^a^	
		Global *p*	Channels (p < .05)	Global *p*	Channels (*p* < .05)
Absolute power	Theta			.16	C4
	Alpha	.15	F3, F7, T3		
Relative power	Delta			.04	F7, T3
	Theta			.11	F4, C4, T3, Fz
	Alpha	.09	F7		
Mean frequency	Delta	.10	F4, C4, T4, T6, Fz, Cz		
	TOTAL	.02	F3, F7, T3, T5, Fz		

Note: EEG analysis was conducted with N = 97 (^a^ n = 48; ^b^ n = 49).

### Anthropometrics and blood analyses

Differences between the groups in mean corpuscular hemoglobin concentration (MCHC), with higher values in the Active group (Mean = 33.6±1.15) compared with the Passive group (Mean = 32.8±.8; t(90) = 2.1, p = .04, CI .26–1.46), were found. Also, higher mean corpuscular hemoglobin (MCH) values in the Active group (Mean = 31.05±1.5) in comparison with the Passive group (Mean = 30.3±1.9; t(90) = 3.6, p = .001, CI .34–1.2) were observed (see Table 3).

**Table 3:** Group comparisons for anthropometrics and blood analyses.

				t-student (90)			
		Mean (SD)				Confidence Interval 95%	
Variable	Normative Values	Active 48	Passive 44	t	p-level	Inferior	Superior
Triglycerides	< 150	129.05 (58.95)	140.77 (74.27)	-.84	.40	-39.38	15.94
HDL-Cholesterol	> 55	50.70* (13.83)	50.74* (13.95)	-.02	.99	-5.80	5.71
VLDL-Cholesterol	0–40	21.68 (12.33)	25.02 (15.18)	-1.16	.25	-9.05	2.37
LDL-Cholesterol	< 130	120.20 (34.09)	130.09* (34.49)	-1.38	.17	-24.11	4.32
Total-Cholesterol	< 200	192.59 (39.39)	205.86* (39.81)	-1.61	.11	-29.69	3.14
Hemoglobin	12.80–17.40	14.77 (1.20)	14.65 (1.27)	.45	.65	-.40	.64
MCHC	31.60–34.80	33.56 (1.15)	32.80 (.79)	3.61	.001	.34	1.18
MCH	27.10–33.50	31.05 (1.50)	30.31 (1.92)	2.06	.04	.03	1.46
Glucose	< 100	97.91 (15.28)	101.44* (19.24)	-.98	.33	-10.69	3.64
TSH	.35–5.50	2.34 (1.25)	2.58 (1.80)	-.74	.46	-.87	.40
Weight (kg)	—	67.40 (10.29)	66.59 (11.85)	.35	.73	-3.78	5.40
Height (m)	—	1.64 (.10)	1.59 (.10)	2.41	.02	.01	.09
BMI	18.50–24.99	25.00* (3.30)	26.20* (3.50)	-1.69	.09	-2.61	.21

Note: Five subjects from the passive group were not included in this analysis; MCHC = Mean corpuscular hemoglobin concentration, MCH = Mean corpuscular hemoglobin, BMI = Body Mass Index, TSH = Thyroid-stimulating hormone.

* Out of normative range.

## Discussion

In our study, comparisons on cognition, brain electrical activity, and blood variables between physically active and passive healthy seniors were conducted. Significant differences were observed for WAIS scores, where the active group obtained higher scores on the performance IQ, processing speed index, matrix reasoning, digit-symbol coding, and picture arrangement subtests. In addition, the active group showed less EEG delta and theta activity, and more alpha activity than the passive group, mainly in frontotemporal areas. Finally, higher values in the MCHC and MCH variables of the blood testing were observed in the active group. Our findings showing that higher levels of IPA are positively associated with cognition and brain electrical activity are similar to the findings of studies evaluating the influence of structured physical activity [25,27,29,49–52]. It is known that the effects of physical activity on health and cognition depend upon the variation in type, frequency, duration, and intensity of physical activity [23]. Several studies have postulated that old adults can enhance their cognitive performance by practicing aerobic structured physical activity, showing improvements in executive control [52,53], inhibitory control [54], and general cognitive capacity [55]. The effects of structured physical activity on cognitive processes have also been observed in young adults, and these effects are larger when there is an increase of intensity, which generates improvement in attention, cognitive control [49], and processing speed [51]. The presence of similar mechanisms may explain the positive associations of IPA and aerobic structured physical activity with cognition and brain functions. In fact, improvements in perceptual organization subtests associated with higher levels of IPA could be a result of amelioration in visual sequencing that requires the retrieval of visual aspects from the tasks; therefore, visual information may be used for planning, sequencing, and organizing processes, which, in turn, improves task performance [56,57].

Functional changes in the prefrontal cortex have been observed when subjects regularly practice structured aerobic physical activity; these changes are interpreted as adaptations related to improvements in working memory, inhibition, and executive functions [50]. In our study, old adults with higher levels of IPA showed higher left frontotemporal alpha power and lower delta and theta power in the left frontotemporal and right frontocentral areas, respectively, than passive old participants. Additionally, the delta MF in the right frontal, central, and temporal regions and the total MF in the left frontotemporal areas were higher in the active group. These topographical differences are in agreement with previous studies investigating the relationship between structured physical activity and resting EEG [26–31] and are related to the aforementioned cognitive processes [57,58]. The EEG pattern of the passive participants was more similar to the EEG pattern exhibited by individuals who developed cognitive decline seven years after this pattern presentation, according to Prichep et al. [6]; this suggests that IPA could be protective against cognitive deterioration.

To our knowledge, the association of IPA with EEG had not been previously explored despite its great importance, since IPA does not require an additional time expense or special resources like structured physical activity does; IPA is linked to a more active lifestyle in individuals independently of structured physical activity practice.

The physiological mechanisms mediating the relationship of IPA with cognition and EEG may be similar to the mechanisms proposed for aerobic structured physical activity. First, a higher level of aerobic physical activity has an important effect on cardiovascular health [59], which has been related to an increase in both, gray and white matter [60], accompanied by increases in brain activation during cognitive tasks in healthy elderly [20,60]. These cognitive improvements may result from enhanced blood flow following physical activity. Ross and McGuire [32] found that the duration and intensity of IPA were positively associated with the cardiorespiratory function; these results suggest physiological effects of IPA that are similar to the effects of the structured physical activity. Therefore, our results regarding the association of IPA with cognition and resting EEG may be partially explained by the mechanism mentioned above, which is supported by the hematological differences between the groups, i.e., the higher mean concentration of hemoglobin and mean corpuscular hemoglobin in the active group. Notice that no evidence of nutritional deficits across the participants was observed. However, on average, active persons tended to show clinically healthier cardiovascular risk profiles (i.e., lower blood glucose and lower total cholesterol and triglycerides) than passives but showed no significant differences. Clinical criteria are based on the normative ranges; therefore, using a strict clinical criterion, the mean values of LDL-Cholesterol, Total-Cholesterol, and Glucose in the passive group were above these limits; while in the active group, these mean values were within the normative limits.

A limitation of the present study could be the use of a self-reported survey to assess the level of IPA of the participants since it constitutes a subjective measurement. Another weakness is the use of a solely behavioral (psychometric) test to evaluate cognition; instead, psychophysiological tools, such as event-related potentials, should be utilized to conduct further studies. Considering the complexity of the physiology of physical activity, future research may be conducted to investigate other physiological mechanisms associated with the benefits of IPA, such as neurotrophic factors [61] or neuroprotective effects [62].

The relevance and novelty of this study rely on unraveling the importance of IPA without detracting the benefits that structured physical activity provide to health and cognition in old adults. Hence, the increase of incidental physical activity may be an alternative to structured physical activity when environmental, personal or motivational conditions hinder the practice of the latter.

## Conclusion

This study presents, for the first time, an analysis of the association between incidental physical activity, cognitive performance, resting EEG, and anthropometric and blood variables in old adults. The obtained results show that incidental physical activity is associated with better scores on cognitive tasks, an increased EEG frequency, and better physiological status, which suggests that incidental physical activity may be protective against age-related deterioration.
